# Physical activity patterns during pregnancy through postpartum

**DOI:** 10.1186/1472-6874-9-32

**Published:** 2009-11-19

**Authors:** Katja Borodulin, Kelly R Evenson, Amy H Herring

**Affiliations:** 1 University of North Carolina at Chapel Hill, Gillings School of Global Public Health, Department of Epidemiology, Chapel Hill, North Carolina, USA; 2 National Institute for Health and Welfare, PO Box 30, FI-00271, Helsinki, Finland; 3 University of North Carolina at Chapel Hill, Gillings School of Global Public Health, Department of Biostatistics, Chapel Hill, North Carolina, USA

## Abstract

**Background:**

Realizing the importance of regular physical activity, particularly in the prevention of chronic diseases and unhealthy weight gain, it is important to study how physical activity changes during and after pregnancy using prospective study designs. The aim of this study was to describe the mode, duration, intensity, and changes in physical activity during pregnancy through one year postpartum among a cohort of women.

**Methods:**

This study was part of the third Pregnancy, Infection and Nutrition Postpartum Study at the University of North Carolina Hospitals. A cohort of 471 women was followed at 17-22 and 27-30 weeks' gestation and at 3 and 12 months postpartum. The participants reported the mode, frequency, duration, and intensity of all physical activities that increased their breathing and heart rate in the past week.

**Results:**

Overall physical activity for the cohort decreased from 17-22 weeks to 27-30 weeks of gestation, but rebounded up at 3 months postpartum and remained stable at 12 months postpartum. The mean MET h/wk values for each time point were 24.7 (standard deviation, SD 26.8), 19.1 (SD 18.9), 25.7 (SD 29.3), and 26.7 (SD 31.5). In postpartum, women reported more care-giving and recreational activity and less indoor household activity, as compared to their activity level during pregnancy.

**Conclusion:**

For health benefits and weight management, health care professionals are encouraged to provide pregnant and postpartum women with information on recommendations of physical activity, particularly regarding the minimum duration and intensity level.

## Background

For health benefits, regular physical activity is recommended for pregnant and postpartum women [1,2]. Previous prospective studies have reported lower levels of recreational, occupational, and overall physical activity during pregnancy [3-5]. However, less is known about physical activity during postpartum and the change in activity from pregnancy to postpartum. Prospective and retrospective studies have suggested, somewhat inconsistently, decreased [6,7], increased [8-11], or unchanged [12,13] physical activity at postpartum as compared to pre-pregnancy or pregnancy levels. Walking is reported to be the most common type of activity during pregnancy [6,14-16] and may remain unchanged from pre-pregnancy to postpartum [8].

Previous studies often collected only recreational physical activity [6-9,11-13], while only a few studies have reported changes in other modes of activity [9,12,13] or in overall physical activity [10,13]. Studies reporting total activity levels have been limited to small, selected samples [10,13] or have measured only pre-pregnancy activity levels instead of pregnancy levels [13]. Physical activity during and after pregnancy includes a variety of activities other than just recreational activity. For example, at one year postpartum, one study found that only 3% of the day was spent on walking, shopping, cycling, or sport activities and the remaining time was spent on various other activities, such as household and care-giving [10]. How the total levels change from pregnancy to postpartum has not been explored in depth.

Realizing the importance of regular physical activity, particularly in the prevention of chronic diseases and unhealthy weight gain [1,17], it is important to study how physical activity changes during and after pregnancy using prospective study designs [18]. The aim of this study was to describe the mode, duration, intensity, and changes in physical activity during pregnancy through one year postpartum in a cohort of 471 women. Past week physical activity was measured similarly at 4 different times; at 17-22 weeks' and 27-30 weeks' gestation as well as 3 and 12 months postpartum. We hypothesized that total physical activity would decline towards the end of pregnancy and increase by 12 months postpartum, with a shift in the types of physical activity from recreational and occupational activity towards care-giving and household oriented activity.

## Methods

This study was part of the third Pregnancy, Infection, and Nutrition Study (PIN3) and the PIN Postpartum Study. Together these studies represent a prospective examination of the association of physical activity and stress with preterm birth and postpartum weight retention. A cohort of women was recruited at prenatal clinics at the University of North Carolina Hospitals, North Carolina, if they were less than or equal to 20 weeks' gestation. Exclusions for the PIN3 recruitment included women under the age of 16 years, non-English-speakers, those not planning to continue care and deliver at the study site, women carrying multiple gestations, and women who did not have a telephone from which they could complete phone interviews. Recruitment began in January 2001 and ended in December 2005. The study protocol was approved by the University of North Carolina Institutional Review Board and participants gave their written informed consent. More detail on the cohort study is available elsewhere http://www.cpc.unc.edu/projects/pin.

During pregnancy, women participated in two telephone interviews at 17-22 and 27-30 weeks' gestation. In postpartum, women completed in-home interviews at approximately 3 and 12 months postpartum (median time of the interview was at 3.9 and 12.9 months). During the 17-22 week telephone interview women reported their race and ethnicity, marital status, education, parity (live plus still births), general health status, and of any bedrest during pregnancy. Self-reported height and pre-pregnancy weight were collected at the first prenatal visit or at the time of recruitment and were used to calculate pre-pregnancy body mass index (BMI, kg/m^2^). Pre-pregnancy BMI was grouped according to the Institute of Medicine's (1990) recommendations in effect during the study period: underweight (BMI < 19.8), normal weight (BMI 19.8-26.0), overweight (BMI 26.1-29.0), and obese (BMI > 29.0)[19].

### Physical Activity Measurement

Interviewers administered a past-week recall on physical activity during telephone and in-home interviews, at pregnancy and postpartum, respectively. The physical activities captured included recreational, occupational, transportation, child and adult care-giving, indoor household, and outdoor household activity that increased breathing and heart rate. For each activity, the participant reported the number of sessions per week, duration of each session, and the perceived intensity level using the following options: 'fairly light,' 'somewhat hard,' and 'hard or very hard.' In addition, the activities were later assigned an absolute intensity level using published metabolic equivalent (MET) tables [20,21], in which one MET corresponds to energy expended at rest per hour/kilogram [22]. The outcome variables were hours (h/wk) and MET-hours (MET h/wk) in the past week. The detailed measurement of activity is reported elsewhere [23]. In separate validation and reliability studies, the questionnaire has shown substantial agreement for test-retest reliability and from low to substantial agreement for criterion validity [23,24].

### Statistical methods

Poisson regression models with generalized estimating equations (GEE) with compound symmetry working correlation [25,26] for repeated count measures were applied to test whether the significant changes occurred in physical activity (total or by intensity levels) across the 4 time points. The goodness-of-fit statistics of all models indicated over dispersion; therefore the Pearson scaling adjustment was applied with compound symmetry as the working correlation structure. All GEE models were adjusted for potential confounders: age (16-25, 26-34, >= 35 years), race/ethnicity (Non-Hispanic white, Non-Hispanic black, other), education (< = 12, 13-15, >= 16 years), marital status (partnered or not), parity (0, 1, 2 or more), general health status (excellent or very good, good, fair or poor), and pre-pregnancy BMI (underweight or normal weight, overweight or obese). Further adjustment for bedrest during pregnancy did not meaningfully change the results; thus it was not included in the final models. The SAS statistical package was used (version 9.1, SAS Institute Inc., Cary, NC).

## Results

### Study sample

During the study period from 2001 to 2005, among the 3203 women eligible for the study, 2006 were successfully recruited and provided informed consent. Of the 2006 women who were enrolled for the PIN3 Study, 37 had multiple birth or lost pregnancy, 159 requested to drop out or no future contact, 84 did not complete the first PIN3 interview, 87 did not deliver at UNC Hospitals or moved out of the area, 8 had medical problems at delivery, and 462 delivered before the Postpartum Study began. Thus, 1169 women were eligible for the postpartum recruitment. A total of 231 were then excluded for the following reasons: 24 had medical constraints, 153 were unreachable, and 54 were greater than 5 months postpartum when we contacted them. Of the 938 women who were asked to participate, 688 (73.3%) agreed to participate and completed a three-month home interview. There were no significant differences (p < 0.05) between the women who completed the 3-month interview and those that were excluded or refused, accounting for women enrolling more than once, for any of the socio-demographic characteristics (age, race/ethnicity, marital status, education), as well as pregravid BMI, parity, bedrest, general health, and total physical activity (in both h/wk and MET h/wk).

Five hundred and fifty (79.9%) of the participants completing the 3 month postpartum visit went on to complete the 12-month component. Participants who became pregnant between the two postpartum time points (n = 45) and those who moved out of the recruitment area (n = 73) were not eligible for the 12-month home visit, thus explaining most of the attrition. The remaining 20 women were excluded for medical complications or were lost to follow-up. After excluding women who participated in the PIN3 Study for a second or third time (n = 64) or had missing information on physical activity variables (n = 15), the final sample comprised 471 women that completed interviews at all 4 time points.

Approximately three-fourths (77.6%) of the women in our sample were non-Hispanic white and 84.1% were married or partnered (Table 1). For about half of the women (54.6%), this was their first pregnancy. The mean education levels were relatively high (16.1 years), as was perceived general health. In the sample, about one-quarter (28.5%) were classified as overweight or obese. Few women reported being advised to stay on bed rest and most women reported at least some moderate to vigorous physical activity.

**Table 1:** Characteristics of the study participants (n = 471).

	% or mean (SD)
Age at the start of pregnancy (mean years, SD)	29.9 (5.4)
Race and ethnicity (%)	
Non-Hispanic white	77.6
Non-Hispanic black	12.1
Other	10.2
Marital status at pregnancy (%)	
Married or partnered	84.1
Single/separated/divorced/widowed	15.9
Parity at the start of pregnancy (%)	
No children	54.6
One child	31.6
Two children	10.2
Three or more children	3.6
Education (mean years, SD)	16.1 (2.6)
≤ 12 years of education	12.9
13-15 years of education	17.2
≥ 16 years of education	69.9
Reported any physical activity (%)*	
17-22 weeks' gestation	96.2
27-30 weeks' gestation	92.1
3 months postpartum	91.3
12 months postpartum	91.9
Perceived general health at 17-22 weeks' gestation (%)	
Excellent	34.6
Very good	44.2
Good	18.3
Fair or poor	3.0
Bedrest required during pregnancy (%)	
Yes	4.7
No	95.3
Pre-pregnancy body mass index (%)	
Under weight	13.6
Normal weight	57.9
Overweight	10.0
Obese	18.5

* Includes any reported recreational, occupational, transportation, child and adult care giving, indoor household, and outdoor household physical activity.

Values are percentages or means (with standard deviations, SD).

**Table 2:** Means (standard deviations, SD) and medians (interquartile ranges, IQR) of overall activity^a ^at four different time points (n = 471)

	**17-22 weeks' gestation**		**27-30 weeks' gestation**		**3 months postpartum**		**12 months postpartum**	
	
	**Mean (SD)**	**Median (IQR)**	**Mean (SD)**	**Median (IQR)**	**Mean (SD)**	**Median (IQR)**	**Mean (SD)**	**Median (IQR)**
	
Fairly Light (h/wk)	3.9 (5.0)	2.3 (0.8-5.0)	3.4 (4.5)	2.2 (0.5-4.5)	3.8 (5.9)	1.6 (0.4-4.7)	3.4 (5.3)	1.8 (0.3-4.3)
Somewhat Hard (h/wk)	2.3 (4.8)	1.0 (0.0-2.7)	1.7 (3.0) *	0.8 (0.0-2.1)	2.7 (5.0) *	1.0 (0.0-3.4)	2.6 (4.6)	1.0 (0.0-3.0)
Hard or Very Hard (h/wk)	0.5 (2.0)	0.0 (0.0-0.0)	0.3 (1.4)	0.0 (0.0-0.0)	0.5 (1.5) *	0.0 (0.0-0.0)	0.7 (2.2)	0.0 (0.0-0.0)
Total (h/wk)^b^	6.6 (7.4)	4.4 (2.1-8.0)	5.4 (5.4) *	4.0 (2.0-6.8)	7.0 (8.2) *	4.2 (1.7-9.0)	6.7 (7.6)	4.2 (1.9-8.7)
Total (MET h/wk)^c^	24.7 (26.8)	17.2 (8.1-31.9)	19.1 (18.9) *	14.3 (6.5-27.0)	25.7 (29.3) *	15.9 (6.0-34.6)	26.7 (31.5)	18.3 (6.3-33.3)

* Significantly different from the preceding time point at alpha level p < 0.05 (GEE model, adjusted for age group, race/ethnicity, education, marital status at pregnancy, parity, general health status, and pre-pregnancy body mass index).

^a ^Overall activity included recreational, occupational, transportation, child and adult care-giving, indoor household, and outdoor household activity.

^b ^Hours per week (h/wk); total h/wk is a sum of fairly light, somewhat hard, hard, or very hard activity.

^c ^MET hours per week (MET h/wk); total MET h/wk is the sum of total h/wk multiplied by established MET value^19,20 ^of the reported type of physical activity.

### Change in the modes of physical activity

The total h/wk and MET h/wk of physical activity decreased from 17-22 weeks to 27-30 weeks of gestation, but rebounded up at 3 months postpartum and remained stable at 12 months postpartum among this cohort of women (Table 2). The mean MET h/wk values of total activity levels for each time point were 24.7 (standard deviation, SD 26.8), 19.1 (SD 18.9), 25.7 (SD 29.3), and 26.7 (SD 31.5) and corresponding median values were 17.2 (interquartile range, IQR 8.1-31.9), 14.3 (IQR 6.5-27.0), 15.9 (IQR 6.0-34.6), and 18.3 (IQR 6.3-33.3). A similar pattern of decreasing total activity during pregnancy and rebounding back at postpartum was observed for somewhat hard intensity activities. The hard or very hard intensity activities remained stable during pregnancy, but increased to 3 months postpartum and remained unchanged thereafter. These observed changes were statistically significant, even when adjusting for age, race/ethnicity, education, marital status, parity, general health status, and pre-pregnancy BMI. A large part of total physical activity consisted of fairly light physical activity, which remained stable across the 4 time points, with mean h/wk values of 3.9 (SD 5.0), 3.4 (SD 4.5), 3.8 (SD 5.9), and 3.4 (SD 5.3).

**Figure 1:**
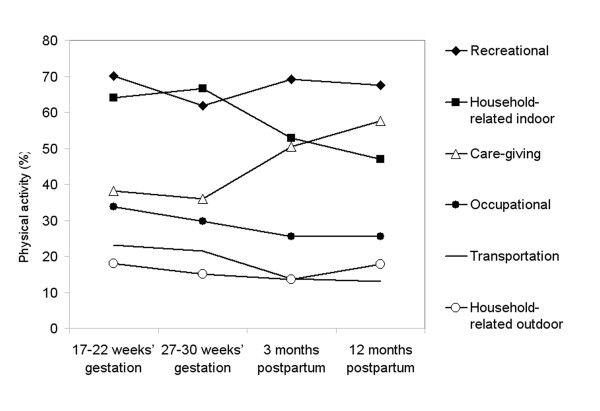
**Participation (%) in different modes of physical activity at 17-22 and 27-30 weeks' gestation and 3 and 12 months postpartum in PIN3 Postpartum Study**. Participation in each mode is defined as report of any activity separately for each of the time points (n = 471).

The percentage of women reporting care-giving, outdoor household, recreation, transportation, and occupational activity decreased from the 17-22 weeks to 27-30 weeks of gestation, with an exception of increased percentage in household-related indoor activity (Figure 1). Participation in any household-related indoor activity decreased from the pregnancy level of 64% at the 27-30 weeks' gestation to 47% at 12 months postpartum. In postpartum, the largest increase from pregnancy levels was reported for care-giving activity, from 38% at the 17-22 weeks' gestation to 51% at 3 months postpartum and to 58% at 12 months postpartum. The highest and most stable participation was reported for recreational physical activity, with around 67% participating during all 4 measurement periods.

### Proportion of different modes contributing to total activity

**Figure 2:**
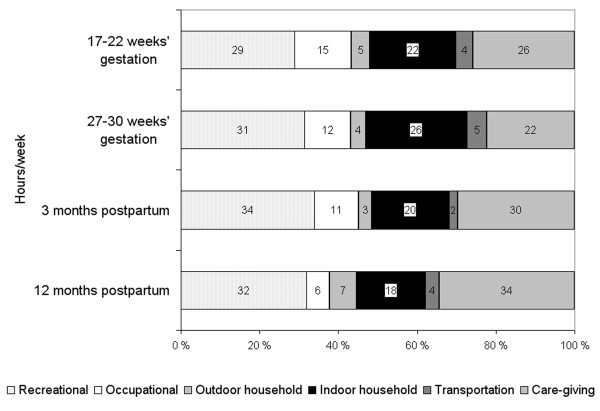
**Proportion (%) of modes of physical activity that contributed to overall activity in hours per week at 17-22 and 27-30 weeks' gestation and 3 and 12 months postpartum in PIN3 Postpartum Study (n = 471)**.

The separate activity modes that contributed the most to the total amount of physical activity in h/wk were care-giving and recreational activities (Figure 2). Care-giving physical activity constituted 26% of total activity level at the 17-22 weeks' gestation, decreased to 22% at the 27-30 weeks' gestation, but increased thereafter to 30% at 3 months postpartum and to 34% at 12 months postpartum. Recreational physical activity contributed around 31% to total activity and remained fairly stable from pregnancy through postpartum.

## Discussion

These cohort data on pregnant and postpartum women suggest that overall levels of physical activity decreased from the 17-22 weeks to 27-30 weeks of gestation, but rebounded up at 3 months postpartum and remained stable at 12 months postpartum. Most of the activities were of lower intensity. Participation in most modes of physical activity decreased during pregnancy, while in postpartum the changes in the activity modes were more varied, with marked increases in care-giving activity and decreases in indoor household activity. Care-giving and recreational physical activity constituted the largest proportion of total activity levels.

### Comparisons to previous scientific literature

One study [10] measured overall physical activity from pregnancy to postpartum, but included only 25 women and did not report activity across intensity levels. Those results suggested, in line with our findings, decreased activity during pregnancy and a rebound at one-year postpartum. They also estimated that at one-year postpartum, 34% of daily energy expenditure was spent on sleeping or lying on a sofa, 35% on sitting, 28% on light household and care, and only 3% on walking, cycling and other sport activities. They did not report the change in the proportions of modality during pregnancy through postpartum. Another study [13] among 63 women reported no change in total activity and measured activity only at pre-pregnancy and 2 and 7 months postpartum, making comparisons to our study difficult.

The percentage of women reporting any activity was the highest and most stable over time for any recreational physical activity, with values of 70%, 62%, 69%, and 68% for the 4 time points. Previous studies [14,15,27-29] have not included comparisons of different modes of activity. Nevertheless, walking is the most commonly reported form of recreational activity during pregnancy [6,14-16]. Two prospective studies have reported decreased percentages in exercise or sports from 63% to 39% [27] and from 72% to 37% [28] from pre-pregnancy to pregnancy. One retrospective study reported a change in the percentages from 48% to 42% [29] for exercise from pre-pregnancy to pregnancy and another study [15] reported fairly stable percentages of 81%, 76%, 73%, and 66% for recreational activity and 70%, 65%, 57%, and 49% for structured exercise from pre-pregnancy through all trimesters. One cross-sectional study [14] using a representative sample of the U.S. population reported that 66% of pregnant women engaged in any leisure time physical activity.

### Methodological considerations

Several limitations should be acknowledged. Physical activity measurement in our study was based on self-reports via interviews, which is not as accurate a measurement method as objective methods, such as accelerometers [30]. However, it allowed for the characterization of the types of physical activities, not yet easily detected with accelerometry. Self-reported physical activity measurements can be prone to measurement error and misclassification, which most often incur bias towards the null. To minimize this bias, a seven day recall was selected instead of a longer recall time period and the assessment was done by a trained interviewer instead of using self-administration [22]. The recall was developed for this study and the test-retest reliability and validity were tested in pregnant women [23,24], but has not been confirmed in postpartum women. The physical activity questionnaire was conducted by telephone during pregnancy and at in-home visits during postpartum, which may have resulted in bias due to the different interview contexts. Furthermore, this physical activity questionnaire was not administered before pregnancy. Thus we cannot speculate whether the women returned to their earlier activity habits after the baby was born.

A commonly reported concern in clinic-based pregnancy cohort studies is the loss to follow-up [31,32]. The preceding PIN1 and PIN2 studies reported that underrepresented women enrolled during early pregnancy were most often lower educated, younger, African American, had higher parity, and had a higher pregnancy risk profile [31,32]. For the PIN3 study, we were unable to characterize differences between those who initially enrolled in pregnancy and those who did not. However, we found no meaningful differences between the women who completed the 3-month interview and those who were excluded or refused to complete the 3-month interview. Even so, we cannot rule out the potential for selection bias and reduced generalizability.

## Conclusion

It is promising that many women returned to their early pregnancy physical activity routines already at 3 months postpartum. Most of the activities during the four measured time points, however, were of lower intensity, which may not be enough to gain health benefits or support weight management. As hypothesized, women reported more often care-giving physical activity in postpartum than during pregnancy and care-giving physical activity also constituted the largest proportion of total physical activity in postpartum. Thus, prenatal and neonatal practitioners are encouraged to provide pregnant and postpartum women with tailored information on the health benefits and recommendations of physical activity during and after pregnancy.

## Competing interests

The authors declare that they have no competing interests.

## Authors' contributions

KB served as the leading author, participated in the planning of the manuscript, and carried out some of the statistical analyses. KRE and AHH participated in the design and coordination of the study and helped in planning, drafting, and finalizing of the manuscript. All authors read and approved the final manuscript.

## Pre-publication history

The pre-publication history for this paper can be accessed here:

http://www.biomedcentral.com/1472-6874/9/32/prepub
